# Brain perfusion estimation by Tikhonov model-free deconvolution in a long axial field of view PET/CT scanner exploring five different PET tracers

**DOI:** 10.1007/s00259-023-06469-w

**Published:** 2023-10-16

**Authors:** Henrik Bo Wiberg Larsson, Ian Law, Thomas L. Andersen, Flemming L. Andersen, Barbara M. Fischer, Mark B. Vestergaard, Tanne S. W. Larsson, Ulrich Lindberg

**Affiliations:** 1 Functional Imaging Unit, Department of Clinical Physiology and Nuclear Medicine, Copenhagen University Hospital-Rigshospitalet, Valdemar Hansens Vej 13, 2600 Glostrup, Denmark; 2 Department of Clinical Medicine, Faculty of Health and Medical Sciences, University of Copenhagen, Copenhagen, Denmark; 3 Department of Clinical Physiology and Nuclear Medicine, Copenhagen University Hospital-Rigshospitalet, Copenhagen, Denmark

**Keywords:** Cerebral blood flow, Long-axial field of view PET, Model-free deconvolution, Extraction fraction

## Abstract

**Purpose:**

New total-body PET scanners with a long axial field of view (LAFOV) allow for higher temporal resolution due to higher sensitivity, which facilitates perfusion estimation by model-free deconvolution. Fundamental tracer kinetic theory predicts that perfusion can be estimated for all tracers despite their different fates given sufficiently high temporal resolution of 1 s or better, bypassing the need for compartment modelling. The aim of this study was to investigate whether brain perfusion could be estimated using model-free Tikhonov generalized deconvolution for five different PET tracers, [^15^O]H_2_O, [^11^C]PIB, [^18^F]FE-PE2I, [^18^F]FDG and [^18^F]FET. To our knowledge, this is the first example of a general model-free approach to estimate cerebral blood flow (CBF) from PET data.

**Methods:**

Twenty-five patients underwent dynamic LAFOV PET scanning (Siemens, Quadra). PET images were reconstructed with an isotropic voxel resolution of 1.65 mm^3^. Time framing was 40 × 1 s during bolus passage followed by increasing framing up to 60 min. AIF was obtained from the descending aorta. Both voxel- and region-based calculations of perfusion in the thalamus were performed using the Tikhonov method. The residue impulse response function was used to estimate the extraction fraction of tracer leakage across the blood–brain barrier.

**Results:**

CBF ranged from 37 to 69 mL blood min^−1^ 100 mL of tissue^−1^ in the thalamus. Voxelwise calculation of CBF resulted in CBF maps in the physiologically normal range. The extraction fractions of [^15^O]H_2_O, [^18^F]FE-PE2I, [^11^C]PIB, [^18^F]FDG and [^18^F]FET in the thalamus were 0.95, 0.78, 0.62, 0.19 and 0.03, respectively.

**Conclusion:**

The high temporal resolution and sensitivity associated with LAFOV PET scanners allow for noninvasive perfusion estimation of multiple tracers. The method provides an estimation of the residue impulse response function, from which the fate of the tracer can be studied, including the extraction fraction, influx constant, volume of distribution and transit time distribution, providing detailed physiological insight into normal and pathologic tissue.

## Introduction

Novel PET/CT scanner designs with a long axial field of view (LAFOV) have a pronounced amplified sensitivity due to a larger solid angle coverage of the patient, resulting in a substantial increase in the possible number of lines of responses (LORs). This gain in sensitivity can be exploited in many ways. One way of utilizing this gain is increasing the temporal resolution in dynamic PET imaging. A higher temporal resolution, i.e. a higher frame rate, is beneficial for the study of dynamic processes such as tracer bolus passage and distribution. Conventional dynamic PET has a frame duration not much lower than 5 s at best, and even a frame duration of 10–20 s can be acceptable to capture a statistically sufficient count number of radioactive events. The mean capillary transit time of the blood is on the order of 1 s, and the vascular mean transit time, i.e. the time it takes for blood to traverse from artery to vein, is of the order of 2–5 s. Therefore, when using conventional PET and PET/CT scanners, it is difficult to capture the fast transient effect of the vascular bolus passage of tracers, which consequently is ignored in the postprocessing of dynamic PET images. Accordingly, tracer kinetic equations do not provide any quantitative information about perfusion and are modified in such a way that only information about the fate of the tracer on a longer time scale is obtained. Only relatively freely diffusible tracers allow estimation of perfusion because tissue transit times are much longer than 1 s, ameliorating the high temporal resolution requirements. For tracers that are not freely diffusible, the initial transient tissue passage, which typically provides information about perfusion and the vascular system in general, is not captured. However, tracer kinetic theory predicts that perfusion can be estimated for all kinds of tracers despite their different fates if temporal resolution is at the level of 1 s or better.

The aim of this study was to investigate whether advanced tracer kinetic methods in combination with high temporal resolution facilitated by new whole-body dynamic PET imaging can provide quantitative information about cerebral blood flow (CBF) and tracer behaviour in tissue, irrespective of whether the tracer is mainly intravascular or freely diffusible across the blood–brain barrier (BBB). Thus, a more generalized and perhaps unbiased analysis of tracer behaviour can be performed. Furthermore, estimation of perfusion, represented by CBF in this study, and compartment rate constants from the same scan using only one type of tracer will allow a more complete interpretation of compartment rate constants in the future and will directly provide information about extraction fractions and the unidirectional influx constant in case of leakage of the tracer across the BBB. Thus, clinical or experimental settings where perfusion and tracer uptake are obtained by two separate PET scans using two different tracers, sequentially or in two separate sessions, can then be reduced to a single dynamic acquisition. As a tangible example, the question of whether a lack of tracer uptake is related to a reduction in perfusion can immediately be addressed with an accompanying CBF map. Finally, the LAFOV PET/CT scanner allows the arterial input function (AIF) to be image derived (IDIF) from the aorta, noninvasively and free of partial volume effects, concurrently with the dynamic imaging of the tissue of interest. When using conventional PET scanners with a limited field of view, an IDIF for neuroimaging will typically be acquired from the carotids or smaller cerebral arteries, which will be contaminated by partial volume effects [1]. This is mitigated when using LAFOV scanners where the IDIF easily can be acquired from purely intravascular voxels in the large aorta.

In this study, we wanted to estimate brain perfusion and the fate of the tracer, characterized by the configuration of the particular tracer’s residue impulse response function, especially the extraction fraction, for the following tracers: ^15^O-labelled water ([^15^O]H_2_O), ^11^C-labelled Pittsburgh compound B ([^11^C]PIB), [^18^F](E)-N-(3-iodoprop-2-enyl)-2β-carbofluoroethoxy-3β-(4′-methyl-phenyl) nortropane ([^18^F]FE-PE2I), 2-deoxy-2-[^18^F]fluoroglucose ([^18^F]FDG) and O-(2-[^18^F]fluoroethyl)-L-tyrosine ([^18^F]FET), ordered by decreasing BBB penetration or increasing diffusion-limited transport into brain tissue. Unsolved aspects of modelling tracer tissue uptake concurrently with tissue perfusion and tracer metabolite conversion will be addressed.

## Theory

The fundamental tracer kinetic equation relates the dynamic tissue time activity curve (TAC) of any tracer to a convolution between the AIF of that tracer and the residue impulse response function (RIF) multiplied by perfusion in the brain, termed CBF [2]. The RIF is defined as the fractional tracer amount in tissue as a function of time after delivery of a short unit bolus into the tissue, mathematically formulated as a delta function. The RIF contains information about the local behaviour of the tracer but is completely free of the assumptions of any underlying tracer kinetic model. The definition is also equivalent to the notion that the RIF represents the time each bolus fraction spends in the tissue, and therefore, the area under the RIF represents the mean transit time (MTT) for the tracer in the tissue [2, 3]. Note that the vascular MTT is much shorter than the MTT comprising the tissue and that the tissue MTT here includes the vascular MTT. We applied a Tikhonov generalized deconvolution (gSVD) method using PET brain data, resulting in CBF, which is a scalar constant, and RIF. It was assumed that the physiological state of the tissue, e.g. CBF, was stable during the measurement. We estimated the brain perfusion of the 5 different PET tracers described above. [^15^O]H_2_O is mainly flow limited in tissue and relatively free to diffuse over the BBB, and RIF is traditionally modelled as a monoexponential function. For [^18^F]FET and [^18^F]FDG, we expected the gSVD method to provide CBF along with additional information about the tissue uptake of the tracer. Both [^18^F]FET and [^18^F]FDG mainly exhibit limited diffusion or transportation in brain tissue and are not freely diffusible over the BBB.

The tissue tracer concentration as a function of time in a region of interest (ROI) or a voxel is related to the AIF, i.e. arterial full-blood tracer concentration, including any metabolites, as a function of time and RIF and scaled by perfusion of the ROI or voxel, by the following equation:

1 $$C_{tis}\left(t\right)=fC_a\left(t\right)\otimes RIF\left(t\right)=\int_0^tC_a\left(t-\tau\right)R\left(\tau\right)d\tau\;\bigwedge\;R\left(t\right)=fRIF(t)$$

where *C*_*tis*_(*t*) is the tissue TAC concentration in an ROI or voxel, *C*_*a*_(*t*) is the AIF obtained from a nearby large artery as an IDIF, *f* denotes perfusion (mL blood min^−1^ 100 mL of tissue^−1^) and RIF(*t*) represents the residue impulse response function, all as functions of time *t* measured in seconds [2]. The integral Eq. 1 is a Volterra integral equation of the first kind and can be considered a Fredholm integral equation with a kernel of zero for *τ* > t. Integration of *C*_*a*_ with *R* is a smoothing operation that results in dampening of the high-frequency components of *R*, while the inverse process of finding *R* from *C*_*a*_ and *C*_*tis*_ tends to amplify the high-frequency components, resulting in a noisy or corrupted R function. Therefore, the solution should be subjected to regularization, or an explicit *R* function type should be chosen based on prior knowledge of the tracer used. The first researcher to employ this convolution formulation in relation to the tracer kinetic theory was Kety [4] in his study of perfusion using a freely diffusible tracer and an explicit expression of RIF as exp(− *f*/*v*_*d*_* t*), where *v*_*d*_ is the volume of distribution (mL 100 mL of tissue^−1^). Substituting this expression into Eq. 1 results in an integral equation that has only 2 free parameters, which can be found through a simple optimization procedure. However, the fundamental assumption in this case is that the RIF behaves as a monoexponential function. More generally, if Eq. 1 is applied to tissue data (ROI or voxel), then the RIF should capture the fate of the tracer irrespective of its distribution in tissue, whether confined to the vasculature or extracted. When estimating the RIF and perfusion without applying any explicit model, a bias-free and more general insight into the fate of the tracer can be assumed. To handle discrete sample data, the integral equation is formulated as a matrix equation as follows:

$$C=\Delta tAR$$

where *A* represents the AIF written as a Toeplitz matrix as follows:

2 $$\left[\begin{array}{c}{C}_{tis}(1)\\ {C}_{tis}(2)\\ {C}_{tis}(3)\\ \vdots \\ {C}_{tis}(n)\end{array}\right]=\Delta t\left[ \begin{array}{c}{C}_{a}(1)\\ {C}_{a}(2)\\ {C}_{a}(3)\\ \vdots \\ {C}_{a}\left(n\right) \end{array}\begin{array}{c}0\\ {C}_{a}(1)\\ {C}_{a}(2)\\ \vdots \\ {C}_{a}(n-1)\end{array}\begin{array}{c}0\\ 0\\ {C}_{a}(1)\\ \vdots \\ { C}_{a}(n-2)\end{array}\begin{array}{c}\dots \\ \dots \\ \dots \\ \ddots \\ \dots \end{array}\begin{array}{c}0\\ 0\\ 0\\ 0\\ {C}_{a}(1)\end{array}\right]\left[\begin{array}{c}R(1)\\ R(2)\\ R(3)\\ \vdots \\ R(n)\end{array}\right]$$

In principle, inversion of matrix A by singular value decomposition (SVD) should directly provide the *R*. However, the matrix equation searching for *R* is a classic example of an ill-posed problem because the A matrix is rank deficient (i.e. a high condition number). Thus, simple SVD results in *R* being dominated by noise. However, stable and meaningful solutions can be obtained through regularization, i.e. by constraining the solution by imposing prior knowledge, such as monotonicity and minimal oscillations. From our definition, the RIF can only stay constant or decrease monotonously from an initial value of 1. As the first step of regularization, *R* is expressed in a B-spline basis as follows:

3 $${R}_{n,1}={B}_{n,k+m}{V}_{k+m,1}$$

where the matrix size is denoted by the subscript, *k* is the number of interior knots and *m* is the order of the B-spline basis. *V*_*k*+*m*,1_ represents the respective B-spline coefficients with *k* + *m* entries. Choosing the order *m* = 4 results in a cubic B-spline basis with continuous first and second derivatives at the knots. We have chosen *k* = *n*/5. The number to be estimated is reduced from *n* to *k* + *m*. The elements of the B-matrix can easily be computed as the Haar basis functions as described in [5]. Substituting Eq. 3 into Eq. 2 results in:

4 $$C=\Delta tAR=\Delta tABV=\Delta tDV$$

Now, the unknown to be estimated is *V* with *k* + *m* entries.

The generalized singular value decomposition by Tikhonov [6, 7] seeks to find the solution *x* of a matrix equation (A*x* = *y*, where *A* and *y* are known, in this case, AIF and *C*_*tis*,_ respectively, and *x* is *R*) from the minimization of the 2-norm of the general form:

5 $${\text{min}}\left\{{\Vert Ax-y\Vert }_{2}^{2}+{\lambda }^{2}{\Vert Lx\Vert }_{2}^{2}\right\}$$

where *L* is a discrete approximation of the first derivative operator as follows:

$$L=\left(\begin{array}{ccccc}-1& 1& \cdots & 0& 0\\ \vdots & \vdots & \ddots & \vdots & \vdots \\ 0& 0& \cdots & -1& 1\end{array}\right)$$

and *λ* is the degree of regularization. If *λ* is set to zero, the solution is not regularized, and *x* may manifest spurious nonphysiological oscillations. If *λ* is set to a very high value, the solution will become oversmoothed without oscillations. The optimum value of the regularization parameter, *λ*, is found using the curvature of the so-called L-curve criteria: a tradeoff between obtaining a smooth *x* and a match between *Ax* and *y*, a criterion developed by Hansen [8]. A practical implementation in perfusion estimation from dynamic contrast-enhanced (DCE) magnetic resonance imaging (MRI) is given in [9].

The following more conventional 2-compartment model with fitted blood volume fraction was also applied to data for comparison. The total tissue concentration (*C*_*tis*_) is:

6 $${C}_{tis}\left(t\right)={V}_{a}{C}_{a}\left(t\right)+(1-{V}_{a}){C}_{t}(t)$$

where *C*_*t*_ is the extravascular tissue concentration composed of a reversible compartment with concentration *C*_*e*_ and a more irreversible compartment with concentration *C*_*i*_ as:

7 $${C}_{t}\left(t\right)={C}_{e}\left(t\right)+{C}_{i}(t)$$

where *C*_*e*_ and *C*_*i*_ are given by the following differential equations:

$$\begin{array}{c}\frac{d{C}_{e}}{dt}={K}_{1}{C}_{a}-\left({k}_{2}+{k}_{3}\right){C}_{e}+{k}_{4}{C}_{i}\\ \frac{d{C}_{i}}{dt}={k}_{3}{C}_{e}-{k}_{4}{C}_{i}\end{array}$$

where *K*_1_ is the unidirectional influx rate constant from blood to tissue, and *k*_2_ is the rate constant from the reversible compartment back to blood, while *k*_3_ is the rate constant related to trapping of tracer in tissue and *k*_4_ is related to a typically very small back leakage from the irreversible compartment.

By use of the Laplace transformation the solution is given by:

8 $${C}_{t}= {K}_{1}{C}_{a}\otimes \frac{\left(\alpha +{k}_{3}+{k}_{4}\right){e}^{\alpha t}-\left(\beta +{k}_{3}+{k}_{4}\right){e}^{\beta t}}{\alpha -\beta }$$

where $\otimes$ denotes convolution and $\alpha$ and $\beta$ are given by:

$$\begin{array}{c}\alpha =\frac{1}{2}\left(-\left({k}_{2}+{k}_{3}+{k}_{4}\right)+\sqrt{{\left({k}_{2}+{k}_{3}+{k}_{4}\right)}^{2}-4{k}_{2}{k}_{4}}\right)\\ \beta =\frac{1}{2}\left(-\left({k}_{2}+{k}_{3}+{k}_{4}\right)-\sqrt{{\left({k}_{2}+{k}_{3}+{k}_{4}\right)}^{2}-4{k}_{2}{k}_{4}}\right)\end{array}$$

Equation 8 is inserted in Eq. 6, and the model is fitted to the observed tissue TAC, where *K*_1_, *k*_2_, *k*_3_, *k*_4_ and *V*_*a*_ are the free parameters. Note that this method does not model perfusion but can theoretically provide an estimate of the blood volume fraction.

## Method

**Fig. 1:**
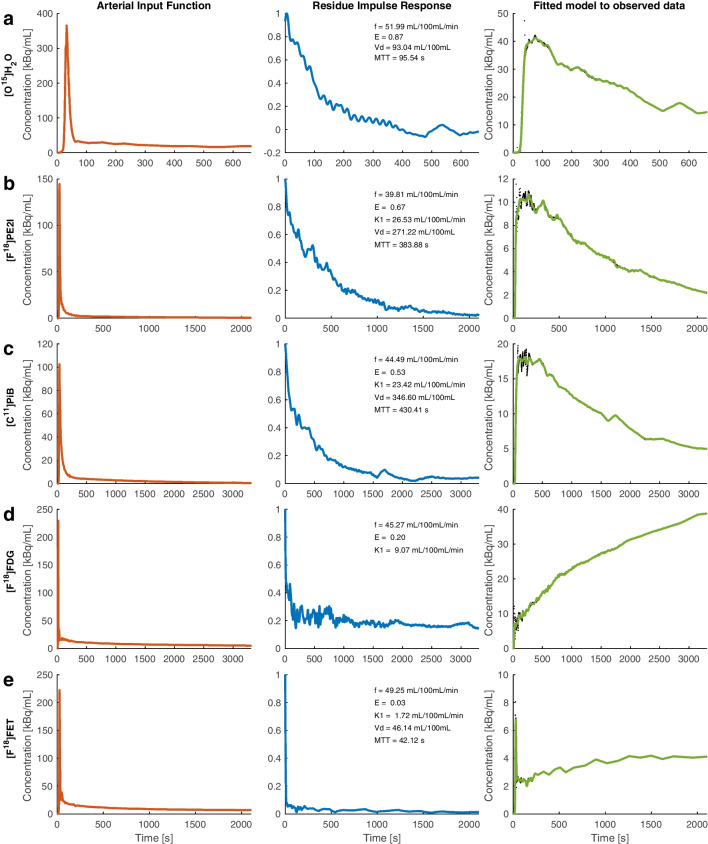
The figure shows results for an ROI in the thalamus in 5 different subjects. Each row corresponds to a different tracer showing the arterial input function (AIF), residue impulse response function (RIF) and model fit to the observed tissue time activity curve (TAC) (black dots: data points including interpolated points; green curve represents the model). The first row (**a**) represents [^15^O]H_2_O. Note the high extraction fraction and a volume of distribution of nearly 1 mL/g. Additionally, note the monoexponential configuration of RIF. **b**: [^18^F]FE-PE2I. Note that the extraction fraction is lower, and the volume of distribution and mean transit time are larger than those of water. **c**: [^11^C]PIB. The extraction fraction is lower, and the volume of distribution is somewhat larger than that of [^18^F]FE-PE2I. For these three tracers, the tissue TAC has an initial maximum and then decays. **d**: [^18^F]FDG. The extraction fraction is much lower, and RIF clearly shows two phases, a fast initial decay that levels off converging towards a constant plateau. The tissue TAC shows a continuous increase, consistent with the configuration of the RIF. **e**: [^18^F]FET. Note the very low extraction fraction; however, it is clearly above zero. Additionally, the tissue TAC curve displays a distinct initial transient, representing the initial tracer bolus in tissue, followed by a steady increase of the tracer in tissue

Twenty-five patients underwent dynamic LAFOV PET scanning on a Siemens Biograph Vision Quadra with a 106 cm field of view (FOV), each with one of the five different tracers. The spatial sensitivity of this type of scanner has previously been published [10]. The sensitivity raises sharply at the entrance of the scanner and about 12 cm from entrance the sensitivity is uniform. The gain in sensitivity is a factor 7–8 compared to state-of-art PET scanner (Siemens Vision 600) and is sufficient to attain a temporal resolution of 1 s. Table 1 shows the number of subjects scanned, and Table 2 shows the dose employed as well as the scanning and reconstruction parameters. The patients scanned with [^18^F]FET were either monitored for a known CNS tumour or suspected of having one. The subjects scanned with [^15^O]H_2_O had steno-occlusive neurovascular disease and were suspected of having reduced vascular perfusion reserve capacity. In accordance with our clinical guidelines, these patients were also studied after an injection of 1–1.5 g acetazolamide (Diamox®). Patients scanned with [^11^C]PIB were clinically evaluated for suspected Alzheimer’s disease, and patients scanned with [^18^F]FE-PE2I were evaluated for suspected parkinsonism. Patients scanned with [^18^F]FDG were examined as part of an oncology whole-body protocol for restaging of cancer or inflammatory diseases. AIF was obtained from the central voxels of the superior descending aorta residing in the arcus aorta with an internal diameter in adults greater than 25 mm, which are not influenced by partial volume effects and are free of adjacent activity spill-in. PET images were reconstructed using low-dose CT for attenuation correction, as shown in Table 2. Time framing was initially 40 × 1 s to secure acquisition of the AIF and tissue TAC peak, with a total scan duration of 12, 40 and 60 min for [^15^O]H_2_O, [^18^F]FET/[^18^F], FE-PE2I, [^11^C]PIB and [^18^F]FDG, respectively (Table 2). Both pixelwise and region of interest (ROI)-based calculations of perfusion were performed using the Tikhonov method by an in-house developed MATLAB (MATLAB version. 9.10.0 (r2021a), Natick, Massachusetts: The MathWorks Inc.: 2021) pipeline utilizing the regularization tools developed by Per Christian Hansen [11]. RIF was used to estimate the extraction fraction (E) of tracer leakage over the BBB by fitting a line to the last part of the RIF curve in a semilogarithmic plot. The unidirectional influx constant *K*_1_ was calculated from the relation *K*_1_ = *E f* [12]. For [^15^O]H_2_O, RIF decays more or less monoexponentially, giving an *E* close to one. The volume of distribution can be calculated as follows:

$$v{_{d}}=\frac{{\int }_{0}^{T}{C}_{tis}\left(t\right)dt}{{\int }_{0}^{T}{C}_{a}\left(t\right)dt}$$

where *T* denotes the total measurement duration. However, for tissue TACs that had not returned to zero at time *T*, the volume of distribution was calculated from the area under the RIF function (giving the MTT of the tracer in tissue) multiplied by CBF, which provides a better approximation for *v*_*d*_. For tracers giving rise to metabolites, some ambiguity exists in the interpretation of *v*_*d*_ (see Discussion). All measured data were upsampled to a 1-s time resolution. No blood sample-based AIF correction for metabolites was performed, as no arterial access was possible in these patients. For [^15^O]H_2_O and [^18^F]FDG, metabolite correction is not needed. [^18^F]FDG is rapidly equilibrated between plasma and the red cell compartment, and in humans, the blood-to-plasma ratio is approximately 0.9 except initially after the bolus injection. On the other hand, it has been shown that the red cell compartment can directly transfer [^18^F]FDG into the brain; therefore, plasma AIF may not actually be better than whole-blood AIF [13, 14]. According to this consideration, no corrections were made for [^18^F]FDG AIF. For [^11^C]PIB, the AIF was corrected by first converting the whole-blood concentration to the plasma concentration using published plasma-to-blood ratio values and for metabolism numerically, according to information in [15] (Figs. 1 and 2 in [15]). In a similar way, [^18^F]FET was metabolite corrected according to information in [16], and finally, [^18^F]FE-PE2I was metabolite corrected according to [17]. For the last 2 tracers, the metabolite correction seemed to be very subtle. For all data, the AIF and the tissue TAC were aligned in time, i.e. the measured time shift provided the arterial transit time delay. This was done for every voxel by identifying the short initial segment of the voxel TAC from scan start to the peak with 10 additional data point (10 s) with corresponding timepoints of the AIF. From these curves a fitting procedure convolutes the AIF with a simple mono-exponential kernel, as *f* exp(− *k*(*t* − ∆*T*)), optimizing *f*, *k* and ∆*T* to fit the short segment of TAC, and where *f* can be interpreted as perfusion, *k* a rate constant and ∆*T* the arterial transit time delay, typically restricted to ± 15 s. The procedure is robust and aligns the AIF and tissue TAC also in noisy situations. ∆*T* was kept and used to align the AIF and tissue TAC before employing the Tikhonov deconvolution voxelvise. The AIF was not dispersion corrected, as justified by the short distance between the arcus aortae and the brain. Data were not motion corrected, but subjects were scanned in a dedicated head fixation holder. Our in-house developed Matlab (version 2021a) pipeline was used for processing the data. As each voxel is being processed individually the use of parallelization greatly reduces the computation time. The more CPU cores available the shorter the computation time. We used a computer with 10 CPU cores for computation. An FDG data set took approximately 2 h to calculate including estimation of the arterial transit time delay.

**Table 1:** Demographics and injected activity

Radiopharmaceutical	*N*	Age [years]	Sex [F/M]	Activity [MBq]
[^15^O]H_2_O	5	66.0 [Range: 56, 76]	2/3	413.6 [IQR: 400, 400]
[^18^F]FE-PE2I	5	77.0 [Range: 62, 83]	2/3	203.2 [IQR: 198, 210]
[^11^C]PIB	5	70.6 [Range: 57, 81]	3/2	301.6 [IQR: 291, 317]
[^18^F]FDG	5	58.6 [Range: 36, 79]	3/2	221.0 [IQR: 199, 266]
[^18^F]FET	5	77.8 [Range: 61, 94]	2/2	196.0 [IQR: 189, 215]

*IQR* interquartile range

**Table 2:** Acquisition and reconstruction parameters

Radiopharmaceutical	Voxel size [mm^3^]	Reconstruction	Framing [s]
[^15^O]H_2_O	1.65 × 1.65 × 1.65	OSEM w/ PSF + TOF 4i5s	40 × 1, 5 × 4, 6 × 10, 3 × 20, 2 × 30, 8 × 60
[^18^F]FE-PE2I	1.65 × 1.65 × 3	OSEM w/ PSF + TOF 4i5s	40 × 1, 10 × 5, 15 × 10, 6 × 60, 10 × 120, 2 × 300
[^11^C]PIB	1.65 × 1.65 × 3	OSEM w/ PSF + TOF 4i5s	40 × 1, 10 × 5, 15 × 10, 6 × 60, 10 × 120, 6 × 300
[^18^F]FDG	1.65 × 1.65 × 2	OSEM w/ PSF + TOF 4i5s	40 × 1, 10 × 5, 15 × 10, 6 × 60, 10 × 120, 6 × 300
[^18^F]FET	1.65 × 1.65 × 3	OSEM w/ TOF 4i5s	40 × 1, 10 × 5, 15 × 10, 6 × 60, 10 × 120, 2 × 300

*OSEM* ordered subset expectation maximum (4 iterations 5 subsets), *PSF* point spread function corrected, *TOF* time-of-flight mode

A conventional compartment model as expressed by Eq. 6 was only applied to tissue TAC ROI’s from thalamus. For all tracers *k*_4_ was restricted to zero, even for [^18^F]FDG, because 1 h of sampling is considered too short to the capture a possible *k*_4_. For [^15^O]H2O, k_3_ was also restricted to zero. The MATLAB function fmincon was used for these calculations.

## Results

**Fig. 2:**
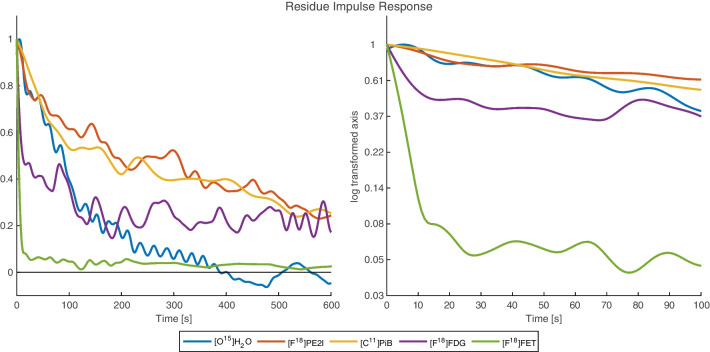
The residue impulse response functions from Fig. 1 depicted on a smaller time scale for better comparison. Both linear and semilogarithmic plots are shown. The configurations of each tracer correspond to the expected behaviour of the tracers

The results are first presented for a region of interest (ROI) in the thalamus using Tikhonov’s procedure for estimation of the integral equation (Eq. 1) for the 5 tracers. For the ROI, we present the AIF, the tissue TAC and the RIF (Fig. 1). Because all subjects were suspected of or suffered from brain diseases, the right or left side was chosen depending on which was the unaffected or least affected side. Furthermore, the thalamus was chosen because this area is free from larger vessels and typically does not accumulate beta amyloid. For [^15^O]H_2_O, the extraction fraction was about 95%, as expected, and RIF decay followed a monoexponential function. For [^18^F]FE-PE2I and [^11^C]PIB, the extraction fractions were 78 and 62%, respectively, which resulted in *K*_1_ values of 45 mL blood min^−1^ 100 mL of tissue^−1^ and 31 mL blood min^−1^ 100 mL of tissue^−1^, respectively. The volume of distribution for the tracers varied greatly, as expected, while CBF was within the expected range. For [^18^F]FDG and [^18^F]FET, a dramatic change in the RIF was observed compared to the RIFs of [^15^O]H_2_O, [^11^C]PIB and [^18^F]PE2I. The RIF exhibited a sharp decrease initially and then levelled off with a slow decrease, somewhat like a biexponential curve. Figure 2 shows the same RIFs as in Fig. 1 but on a smaller time scale for comparison. The initial decrease corresponds to tracer washout from the vascular compartment, while the subsequent slow decrease relates to the extraction of the tracer [2, 3]. The mean extraction fraction for [^18^F]FDG was 19% in the examples we investigated, while the extraction fraction for [^18^F]FET was much lower (in healthy brain tissue) but always clearly discernible. For [^18^F]FET, the tissue uptake curve showed a short transient peak corresponding to the first pass of the tracer entering the tissue. Obviously, this transient peak contains information about perfusion and can easily be missed with a lower temporal resolution. Additionally, in this case, CBF was within the expected range. As shown in Fig. 1, some overfitting of the model to the tissue TAC occurred (see further discussion).

In Table 3, the results of the estimation of perfusion for an ROI in the thalamus are shown.

**Table 3:** Tikhonov method

Radiopharmaceutical	Number of subjects/ROIs	Mean CBF ± SD (mL/min/100 mL)	Mean *E* ± SD	Mean *K*_1_ ± SD (mL/min/100 mL)	Mean *v*_*d*_ ± SD (mL/100 mL)
[^15^O]H_2_O rest	5/5	69 ± 19	0.94 ± 0.06	-	90 ± 5.6
[^15^O]H_2_O acetazolamide	5/5	115 ± 31	0.96 ± 0.04	-	92 ± 6.9
[^18^F]FE-PE2I	5/5	58 ± 15	0.78 ± 0.08	45 ± 16	383 ± 98
[^11^C]PiB	5/5	50 ± 7	0.62 ± 0.08	31 ± 8	288 ± 90
[^18^F]FDG	5/5	37 ± 13	0.19 ± 0.05	5.5 ± 1.2	-
[^18^F]FET	5/5	53 ± 16	0.032 ± 0.008	1.7 ± 0.2	48 ± 9

*CBF* cerebral blood flow, *E* extraction fraction, *K*_*1*_ unidirectional influx constant, *v*_*d*_ volume of distribution

**Fig. 3:**
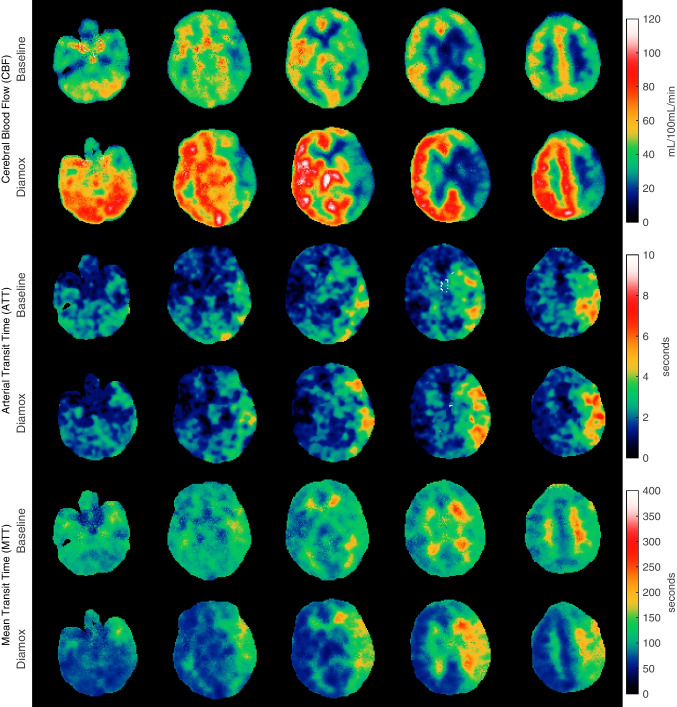
CBF, arterial transit time delay and tissue mean transit time (MTT) maps are shown at rest and after infusion of acetazolamide (Diamox) for a patient suffering from Moya-Moya disease and left internal carotid artery stenosis. Acetazolamide decreases CBF in the affected hemisphere and increases regional arterial transit time delays and MTT. The CBF values are corrected for the regional arterial transit time delays

The pixelwise calculated maps for [^15^O]H_2_O, both at rest and after acetazolamide challenge, are shown in Fig. 3. CBF maps, MTT and arterial transit time delay maps are also shown.

**Fig. 4:**
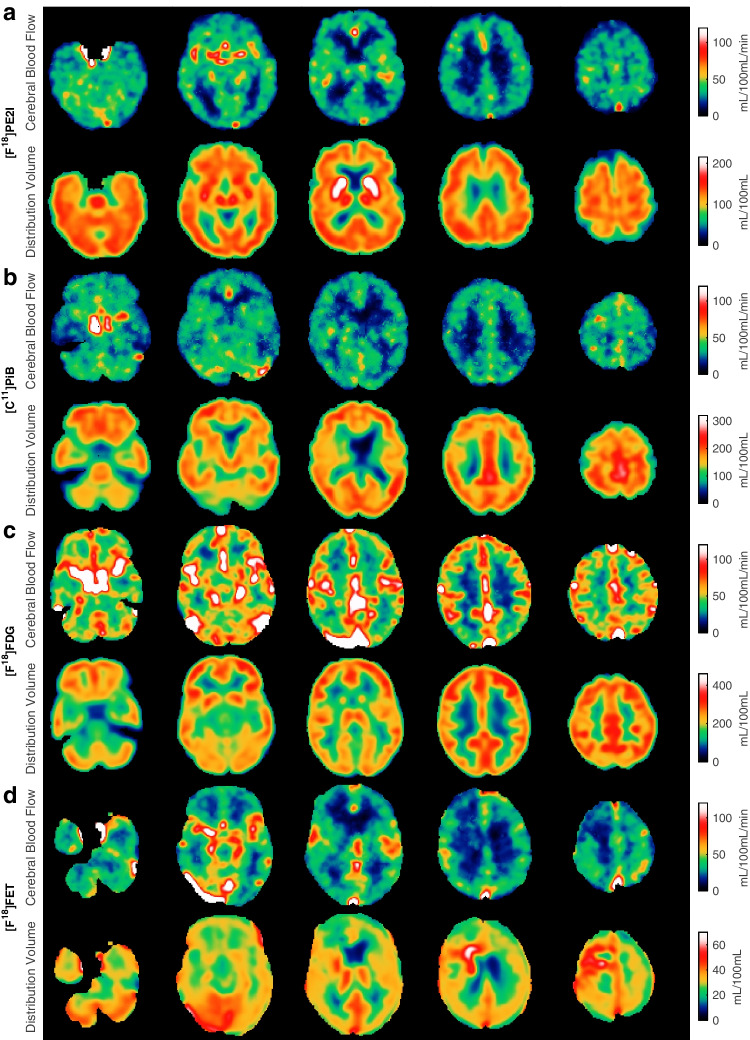
Each row shows the perfusion maps and volume of distribution maps for **a** [^11^C]PIB, **b** [^18^F]FE-PE2I, **c** 2-[^18^F]FDG and **d** [^18^F]FET for four different patients. Patient (a) had Alzheimer’s disease and pronounced beta-amyloid accumulation, and the CBF maps show typical parieto-temporal perfusion reduction (left-sided). Patient (b) was eventually diagnosed with major depression, and the CBF and volume of distribution maps were normal. Patient (c) had lung cancer with metastasis, but PET/CT of the brain did not disclose CNS involvement. Patient (d) had previously undergone surgery for brain cancer (glioblastoma), and the CBF maps show CBF reduction/no perfusion corresponding to the resection cavity, and the volume of distribution map shows abnormal frontal subcortical FET uptake, suggesting tumour recurrence. All images are shown in native orientation to avoid interpolation artefacts

Figure 4a shows an example of [^18^F]FE-PE2I in a patient suspected of having Parkinson’s disease but later diagnosed with major depression. The perfusion maps and the volume of distribution maps are normal. Figure 4b shows the perfusion and volume of distribution maps when using [^11^C]PIB in an amyloid avid patient suspected of having Alzheimer’s disease. The patient showed classic parieto-temporal perfusion reductions with a similar pattern as seen on a conventional static [^18^F]FDG scan of this patient (not shown). Furthermore, the patient had extensive beta-amyloid accumulation seen on the volume of distribution maps. Figure 4c shows an example of [^18^F]FDG in a patient with disseminated non-small cell lung cancer. The PET scan showed no CNS involvement. The distribution between the CBF maps and the volume of distribution maps is similar, which represents the relative distribution of glucose consumption as expected. Finally, Fig. 4d shows an example of [^18^F]FET in a patient previously operated on for a glioblastoma and now having recurrence.

The examples shown are representative but not superior to the other patients scanned. In all cases, CBF maps were produced, but the magnitude of CBF in absolute terms varied, which is partly explained by the patient heterogeneity in disease burden and age.

Table 4 shows results when applying a conventional compartment model to the same ROIs in thalamus.

**Table 4:** Conventional compartment model

Radiopharmaceutical	Number of subjects/ROIs	Mean *K*_1_ ± SD (mL/min/100 mL)	Mean *k*_2_ ± SD (1/min)	Mean *k*_3_ ± SD (1/min)	Mean *V*_*a*_ ± SD (mL/100 mL)
[^15^O]H_2_O rest	5/5	72.6 ± 18.3	0.76 ± 0.17	-	0.88 ± 0.62
[^15^O]H_2_O acetazolamide	5/5	115.7 ± 30.2	1.21 ± 0.27	-	2.07 ± 1.16
[^18^F]FE-PE2I	5/5	47.6 ± 12.3	0.12 ± 0.03	< 0.0004	3.26 ± 2.02
[^11^C]PiB	5/5	41.1 ± 4.6	0.16 ± 0.05	0.003 ± 0.004	4.3 ± 0.82
[^18^F]FDG	5/5	12.8 ± 1.2	0.13 ± 0.03	0.051 ± 0.008	1.93 ± 1.79
[^18^F]FET	5/5	2.9 ± 0.7	0.082 ± 0.02	0.027 ± 0.010	2.94 ± 0.55

*K*_1_ is the unidirectional influx rate constant over the blood–brain barrier, *k*_2_ is a rate constant related to back diffusion of tracer from the reversible compartment to blood, *k*_3_ is a rate constant related to the irreversible binding of tracer in tissue, *V*_*a*_ is the blood volume in tissue

*K*_1_ estimated by the compartment model shows the same decreasing pattern for the tracers compared to Tikhonov’s method, although somewhat higher, especially for [^18^F]FDG and [^18^F]FET. For water [^15^O]H_2_O, *K*_1_ is close to perfusion as expected for a freely diffusible tracer. Interestingly, *k*_3_ seems to be very small for [^11^C]PIB and [^18^F]FE-PE2I and can possibly be omitted from the fit, at least in thalamus, but may become important in other tissue type and in diseases.

## Discussion

This is a proof-of-concept study. Using the high temporal resolution offered by an LAFOV PET scanner in combination with the fundamental tracer kinetic equation (Eq. 1) allows for estimation of tissue perfusion within the expected range for generally accepted perfusion values [18, 19] and the residue impulse response function, providing novel physiological information about the tracer being used. The results show the expected characteristics of the various tracers regarding blood–brain barrier passage, e.g. the degree of extraction fraction that can be obtained concurrently, with values ranging from approximately 0.95 down to approximately 0.03. The method even allows for calculation of voxelwise CBF maps corrected for regional arterial transit delays. At a practical level, specific tracers aiming to explore specific targets in the brain can simultaneously provide information about CBF, thus potentially omitting the need for an additional perfusion PET scan.

The concept of perfusion, especially brain perfusion, is not well defined. One typical way of defining perfusion is the amount of blood entering the capillary segment per unit time and per unit tissue volume. However, perfusion could also be defined as the brain tissue clearance (*K*_1_ or *k*_2_) for a tracer with an extraction fraction of one, e.g. water. The perfusion estimated from Eq. 1 is related to the total amount of blood entering a unit volume of tissue (per unit time) irrespective of vessel topography. Thus, to some extent, the present concept of perfusion is different from, e.g. magnetic resonance arterial spin labelling or PET compartmental modelling using radiolabelled water. Here, perfusion typically does not include tracers in larger vessels, which are considered transport vessels and are not truly involved in tissue perfusion. It should be mentioned that in the MRI community, brain perfusion employing intravascular contrast media is often estimated using the same perfusion concept as in the present study [9, 20–22]. Finally, from a clinical point of view, this discussion might be considered academic because local perfusion abnormalities are discovered anyway. An interesting advantage of using tracers or contrast agents that are confined mainly to the vascular compartment is the ability to probe the vascular architecture, specifically to estimate the distribution of vascular transit times in tissue [23]. This is obviously not possible with freely diffusional tracers, which are classically used for perfusion estimation. The distribution of blood transit times may govern the likelihood of a solute entering the brain tissue. A current hot topic is how the capillary transit time distribution may determine the maximum amount of oxygen delivered to the brain tissue irrespective of local CBF [24, 25].

The importance of accurate temporal alignment of the AIF and tissue TAC is well-known [26, 27]. In conventional [^15^O]H_2_O PET, only one global arterial transit time delay is estimated and applied to all subsequent calculations [26, 27]. The increased temporal resolution provides the ability to estimate local arterial transit time delay, i.e. the time it takes from the measurement location of the AIF to the local tissue site, thus providing additional clinical information, especially in ischaemic brain diseases. In the [^15^O]H_2_O example, an arterial transit time delay of up to 10 s in tissue was observed, and if this delay is not taken properly into account, as with the current standard model, perfusion will be underestimated. Additionally, the tissue mean transit time (MTT) can provide a sensitive clinical parameter in cerebrovascular disease where perfusion reduction leads to tissue vasodilation, and therefore, an increased MTT, i.e. the distribution volume divided by perfusion. This phenomenon is obviously more pronounced for tracers with BBB diffusion restrictions.

From a numerical point of view, Eq. 1 represents a Fredholm integral equation of the first kind where we want to estimate the unknown kernel *R* (*R*(*t*) = *f* RIF(*t*)), which is the solution containing the physiological information. This constitutes a so-called ill-posed problem, having no unique solution, and the estimated solution is very susceptible to small imperfections, e.g. noise, in the measured functions *C*_*a*_(*t*) and *C*_*tis*_(*t*). To stabilize the solution and provide meaningful physiological information, it is necessary to regularize the solution by incorporating the norm of the solution or the norm of the derivative of the solution when solving the equation. This is done by minimizing the total norm in Eq. 4, with balanced reduction between the regularization error ${\Vert Ax-y\Vert }_{2}^{2}$ and the perturbation error ${\Vert x\Vert }_{2}^{2}$ or the first derivative ${\Vert Lx\Vert }_{2}^{2}$. This procedure is referred to as the L-curve criteria [8] and provides an optimum value of the regularization parameter *λ*, which determines the weight of the perturbation error. Setting λ to zero indicates no regularization; the solution results in overfitting with a small regularization error and shows spurious large oscillations with no information. Conversely, choosing a very high *λ* results in an oversmoothed fit to the data, with a large regularization error and a small norm of the solution suppressing all transient excursion. The L-curve provides a balance between these extreme settings. By this procedure, we can suppress the effect of noise in *C*_*tis*,_ which otherwise will be extremely amplified in the solution. An inherent consequence of this procedure is that fast transients, such as the first pass perfusion peak (Fig. 1, [^18^F]FET), tend to be suppressed, which is an unwanted side effect of regularization. This effect is not a serious problem if no such transient phenomenon occurs (Fig. 1, [^15^O]H_2_O, [^11^C]PIB and [^18^F]FE-PE2I). Therefore, the L-curve criteria sometimes tend to suppress the estimated perfusion when using [^18^F]FDG and especially [^18^F]FET. However, as mentioned previously, a tendency towards overfitting is seen. This is an interesting consequence of upsampling tissue TAC to a 1-s time resolution after the initial part that is sampled with a 1-s time resolution; the latter part of the curve has redundant data points and no major transients. The result is that the L-curve optimization procedure finds a smaller regularization value per se, resulting in an overfitting of the tissue TAC. Due to the convolution inherently embodied in Eq. 1, it is very difficult to avoid this interpolation. A further interesting problem is that in our situation, the solution we seek consists of a scale factor (*f*) and the residual impulse response function (RIF), which inherently provides information about the fate of the tracer. Restriction of the norm of the solution can therefore be achieved by either scaling down *f* or excursion of RIF. In this study, we have chosen to use the 2-norm of the first derivative of the solution, resulting in dampening of the spurious oscillation of the solution. Norm reduction could also be based on both the first and the second derivative of the solution and possibly be applied to different timing periods. *K*_1_ estimated from the conventional compartment model was higher compared to estimated *K*_1_ values from Tikhonov’s method. Because Tikhonov’s method provided extraction fraction values in the expected range, it points to perfusion estimated by the Tikhonov’s method is a little underestimated. This again points to the level of regularization. A method to rigorously determine the level of regularization taking global tissue features into account is a subject for an upcoming study.

Perfusion and rate constants have been estimated previously for these tracers, typically employing a 1- or 2-compartment model, occasionally in combination with a separate [^15^O]H_2_O perfusion PET scan for estimation of the extraction fraction. In these situations, the RIF is dictated by the model, and the volume of distribution is typically calculated from the microparameters of the two-compartment model. The correctness of compartment models may be questioned, especially when the dynamic frame rate is fast, as typically used for intravascular and diffusion-limited tracers discussed in the MR literature [20]. For example, compartment models inherently assume a minimum transit time of zero, which obviously is not correct if data are obtained with a time resolution of 1 s or better. [^15^O]H_2_O perfusion is generally modelled with a monoexponential kernel often incorporating the arterial vascular contribution of unextracted tracer into the model [28], as we also did for the conventional method. Our RIF obtained by Tikhonov deconvolution provides a near-perfect monoexponential decay. Future studies should focus on whether a reliable extraction fraction for water can be estimated and whether the extraction fraction changes significantly during an acetazolamide challenge. Several studies have investigated the relationship between the unidirectional blood‒brain transfer constant, *K*_1_, of [^11^C]PIB and CBF, questioning the accuracy of CBF distribution determined by the early uptake of [^11^C]PIB in the brain. These studies are based on a separate measurement of CBF with [^15^O]H_2_O. In the study by Gjedde et al. [29], the authors found an extraction fraction of approximately 55–59% and a *K*_1_ of approximately 25 mL blood min^−1^ 100 mL of tissue^−1^ in patients with Alzheimer’s disease, signifying that [^11^C]PIB uptake is not strictly governed by CBF. We calculated a mean extraction fraction of 62% and a somewhat higher *K*_1_, both for the compartment model and Tikhonov’s method. In principle, our results could be more accurate because our data originate from one PET examination and not 2 separate PET scans. Furthermore, all measurements were performed by the same instrumentation, and the issue of cross calibration of sensitivities is nonexistent. In a [^18^F]FE-PE2I study of rhesus monkeys, Varrone et al. [30] found a volume of distribution in the thalamus of 620 mL 100 mL of tissue^−1^ using intrinsic kinetic parameters, which is 50% higher than our human values. Sasaki et al. [31] also studied [^18^F]FE-PE2I uptake in young healthy men and found a *K*_1_ and volume of distribution in the thalamus of 27 mL blood min^−1^ 100 mL of tissue^−1^ and 380 mL 100 mL of tissue^−1^, respectively, using a two-compartment model. We found a somewhat higher *K*_1,_ while the volume of distribution was similar. Concerning [^18^F]FDG, our results are in good agreement with the results from Huisman et al. [32]. In the thalamus of young healthy men, they found a *K*_1_ of 8 mL blood min^−1^ 100 mL of tissue^−1^ and an *E* of 19% at a CBF level of 46 mL blood min^−1^ 100 mL of tissue^−1^.

The discrepancy between the extraction fraction from our study and those from previous studies using two-compartment models for [^18^F]FE-PE2I and [^11^C]PIB may also be related to the occurrence of metabolites. Our approach relies on an AIF representing whole blood and measuring all tracer activity, including any metabolites, in the tissue time activity curve. Eventually, as metabolites are produced, the free parent plasma tracer concentration decays with its own time constant, and a redistribution between plasma and the erythrocyte compartment and protein binding take place. In many situations, only the parent tracer in plasma is subjected to tissue clearance, complicating the perception of the extraction fraction and the unidirectional influx constant *K*_1_. Therefore, a plasma arterial input function related to the parent tracer concentration is needed for proper estimation of *K*_1_ and the other microparameters. In this study, we used a heuristic approach by incorporating a whole blood-to-plasma ratio time course and a parent plasma tracer decay time course taken from the literature. This was motivated by the fact that these modifications do not change the initial AIF corresponding to the first pass of the tracer bolus in tissue (Fig. 4 in [15]), which is important for perfusion estimation, while tissue accumulation over time will likely be more affected by the later part of the AIF where metabolite development is more pronounced. However, a thorough model of the system can be obtained not by a two-compartment model that inherently ignores perfusion but by a three-compartment model with a blood compartment, a reversible compartment and an irreversible compartment. This system can easily handle whole-blood activity and whole blood-to-plasma redistribution together with tracer metabolite conversion as a function of time (if they are known). The resulting three coupled differential equations can be solved numerically using, e.g. the Runge‒Kutta numerical method estimating 6 parameters, including perfusion. Knowing the perfusion from this procedure may facilitate estimation of the other parameters, a procedure presented previously in an MRI context [22]. It should also be mentioned that a three-compartment model will inherently model the RIF as three-exponential decay (or biexponential decay asymptotically approaching a level above zero, signifying a trapped fraction). For this reason, some ambiguity occurs when we estimate the extraction fraction from our measured RIF.

## Conclusion

We have shown that high temporal resolution offered by the new long axial field of view PET/CT scanners in combination with generalized singular value decomposition—Tikhonov deconvolution—can simultaneously provide reliable estimates of perfusion and the fate of the tracer in the brain irrespective of the blood‒brain barrier permeability of the tracer. The high temporal resolution, together with new tracer kinetic modelling, may also unveil new perspectives of exploring the vascular system and transport between compartments.

## Data Availability

The datasets generated and/or analysed during the current study are available from the corresponding author on reasonable request and collaboration agreement and given approval from relevant regulatory authorities.
